# Supplementary material for: Pennate diatoms make non-photochemical quenching as simple as possible but not simpler
Source: Nat Commun. 2025 Mar 10;16:2385. doi: 10.1038/s41467-025-57298-4 (PMC11894083; doi:10.1038/s41467-025-57298-4)
Supplement: Supplementary file 1 — Supplementary Information [file 41467_2025_57298_MOESM1_ESM.pdf]

Pennate diatoms make NPQ as simple as possible, but not simpler

Dany Croteau, Marianne Jaubert, Angela Falciatore, Benjamin Bailleul*

CNRS, Sorbonne Université, Institut de Biologie Physico-Chimique, Photobiologie et physiologie des plastes et des microalgues, F-75005 Paris, France

*Corresponding author: bailleul@ibpc.fr

Supplementary Information

Figure S1 to S10

Supplementary Text S1 and S2

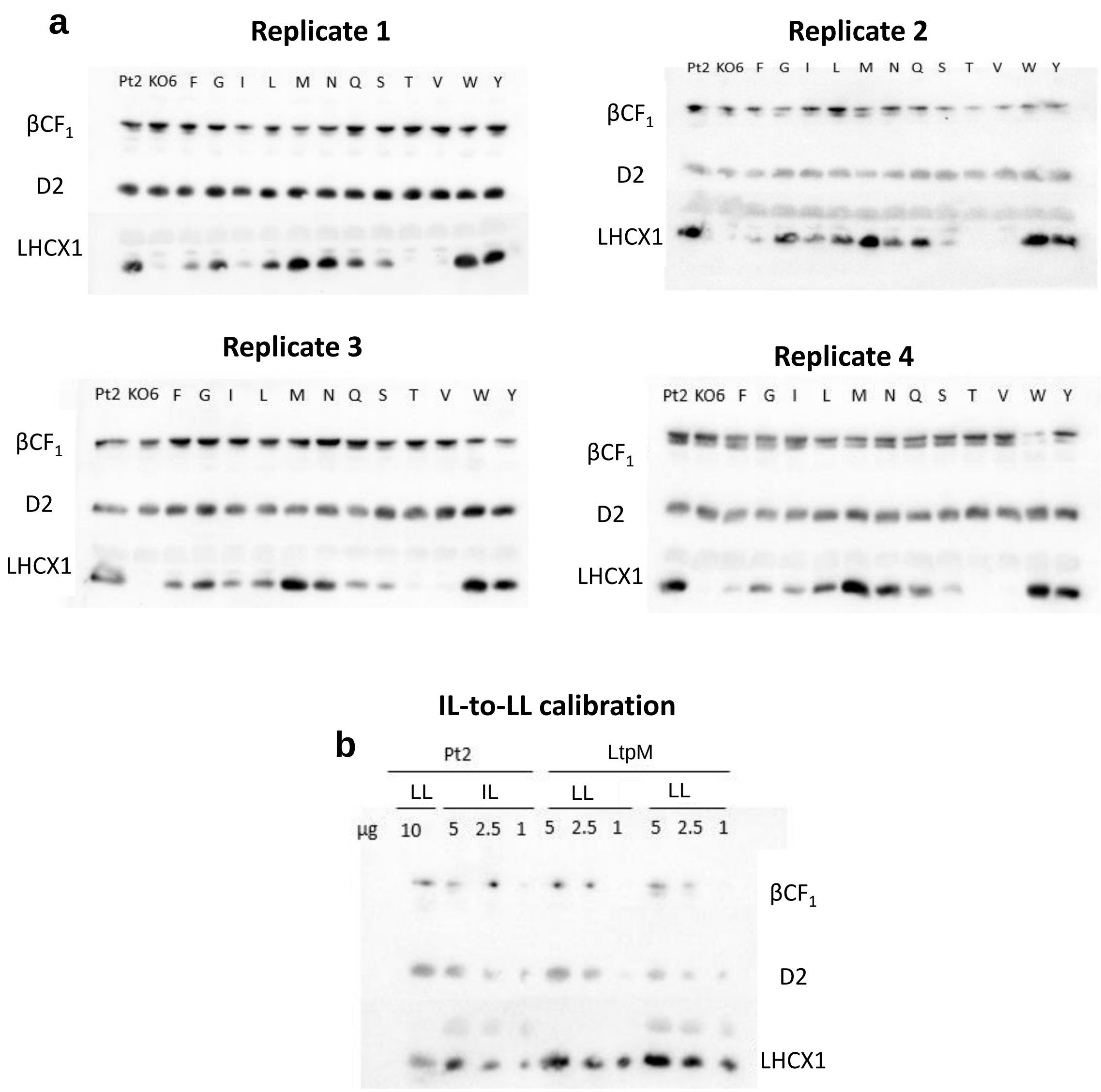

Fig. S1 | Quantification of Lhcx1 by Western blots in *Phaeodactylum tricornutum* strains cultivated under intermittent light (IL) and calibration with pre-existing Lhcx1 quantifications for cultivation under low light (LL).

Four replicates of Western blots for all 14 strains (only the last letter of all complemented lines is shown for clarity purposes) cultivated under IL for quantification of Lhcx1, ATP synthase βCF1 subunit and PSII-D2 subunit (**a**) Western blots for various total protein loadings (between 1 and 10 µg) in wildtype (WT) *Phaeodactylum tricornutum* and complemented strain LtpM cultivated under IL and LL used to calculate the factor of relative [Lhcx1] increase from LL-to-IL (**b**). Raw quantification by Western blots for LL were taken from previous works first published in [1].

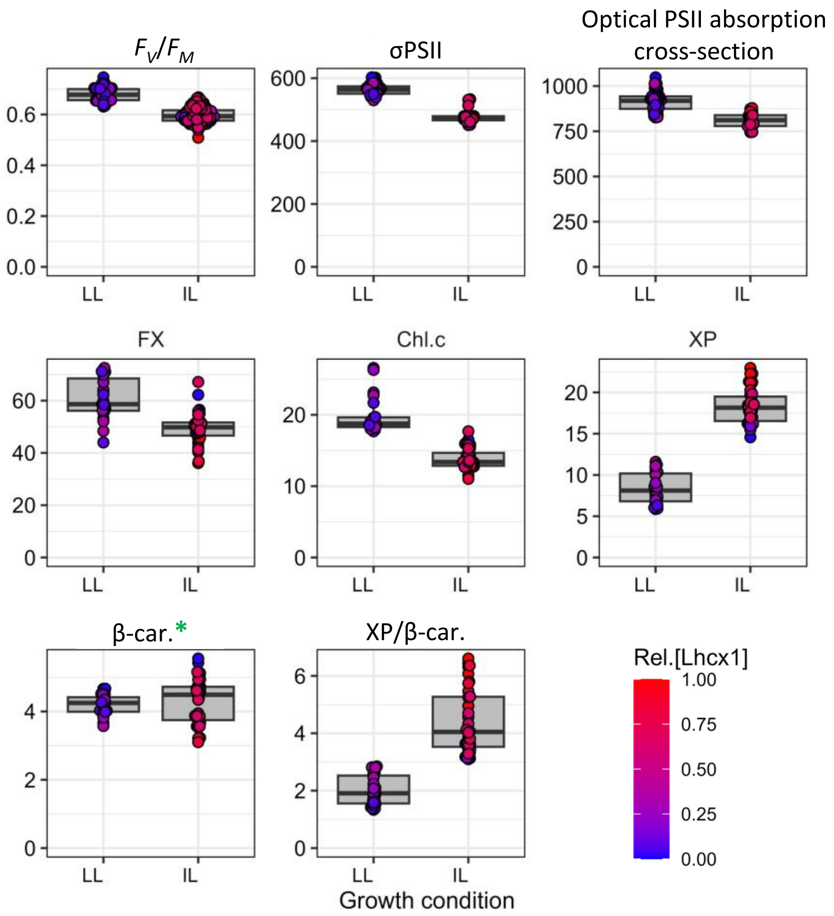

Variable	F_Value	P_Value
Fv/Fm	389.83	2.20E-46
σPSII	286.02	3.40E-24
Optical σPSII	59.30	1.81E-10
FX	36.64	1.50E-07
Chl.c	139.60	2.25E-16
XP	942.77	2.59E-33
β-car.	0.23	6.33E-01
XP/β-car.	363.89	4.93E-24

Fig. S2 | Physiological parameters under low light (LL) and intermittent light (IL) growth conditions in all *Phaeodactylum tricornutum* strains.

Boxplots comparing all values of physiological parameters (excluding Lhcx1 or NPQ-related parameters, see Fig. 1 and Table 1) and pigment content measured across wildtype and all Lhcx1-mutants *Phaeodactylum tricornutum* strains grouped by growth conditions to compare photoacclimation effects (colour scales indicate the relative Lhcx1 concentration in the strain measured (see Fig. 1)). Significant differences between growth conditions were tested using one-way ANOVA with Satterthwaite's method (R software, package lmerTest), accounting for uneven strain numbers and biological replicates. Functional PSII absorption cross-section (σPSII) and optical PSII absorption cross-section are in $Å^2$. Pigment concentrations (fucoxanthin (FX), chlorophyll (Chl) c, xanthophyll pigments (XP, diadinoxanthin+ diatoxanthin), and β-carotene (β-car.)) are normalized to 100 mol. Chl a, except XP/β-car. (mol/mol). The only parameter showing no significant differences (β-car.) is marked with a green star and written in green in the table.

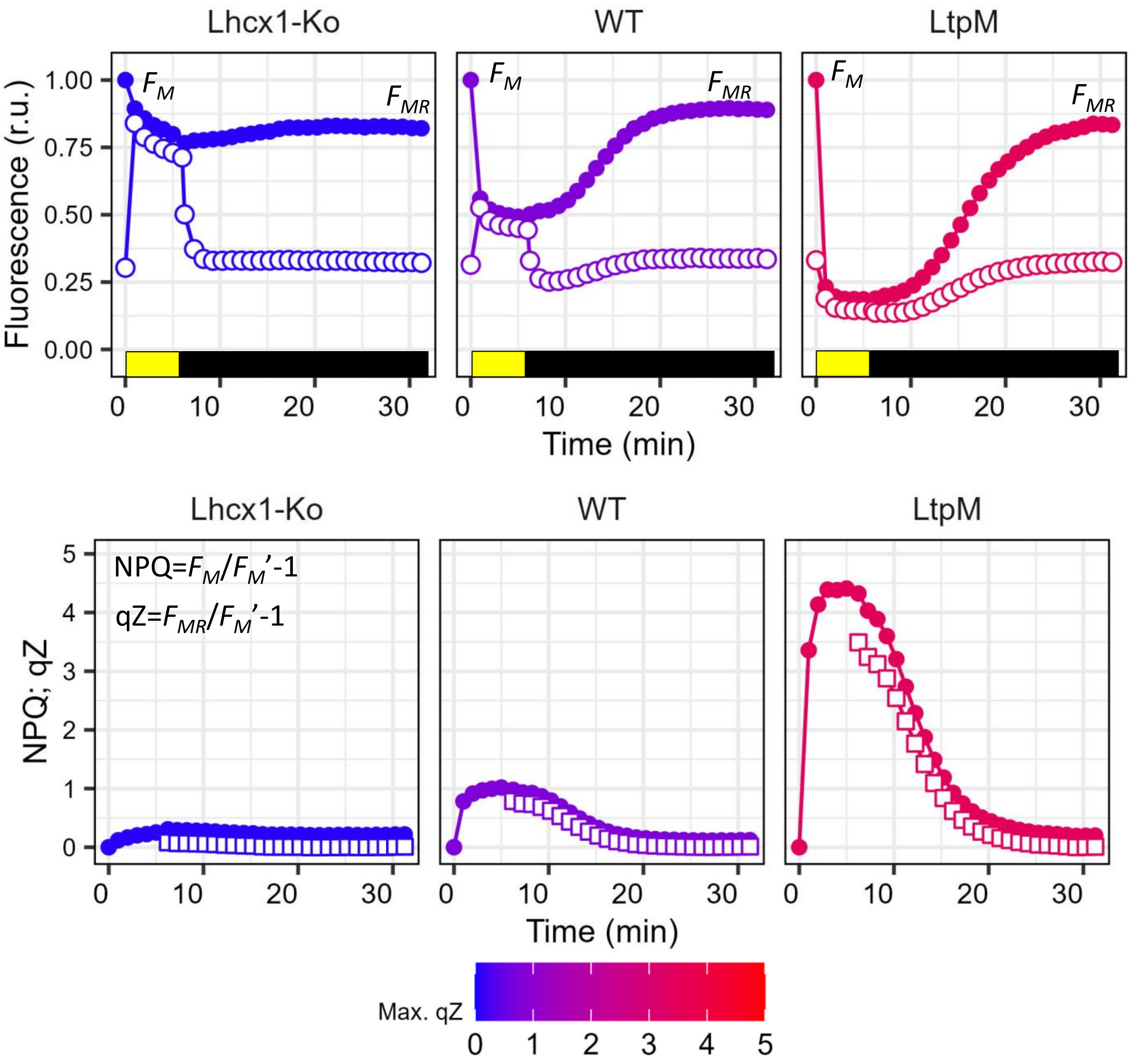

Fig. S3 | Exemplary fluorescence curves and calculation of NPQ and qZ in three *Phaeodactylum tricornutum* strains.

Top row shows maximal fluorescence (closed circles, obtained upon a saturating pulse of light, see Methods) before (F_M), during and after (F_M') a 6 min high light exposure (600 µmol photons m^{-2} s^{-1}) followed by 25 min of recovery in darkness in the Lhcx1-Ko, widltype (WT) and LtpM Lhcx1 overexpressor strains. The fluorescence before the saturating pulse (open circles) was measured before (F_0), during (*F*) and after (F_0') the high light period. Bottom rows shows non-photochemical quenching (NPQ) computed over this same sequence (closed circles) and the rapidly reversible, diatoxanthin-dependent, qZ component of NPQ (open squares, only calculated during the recovery period.

Figure S4

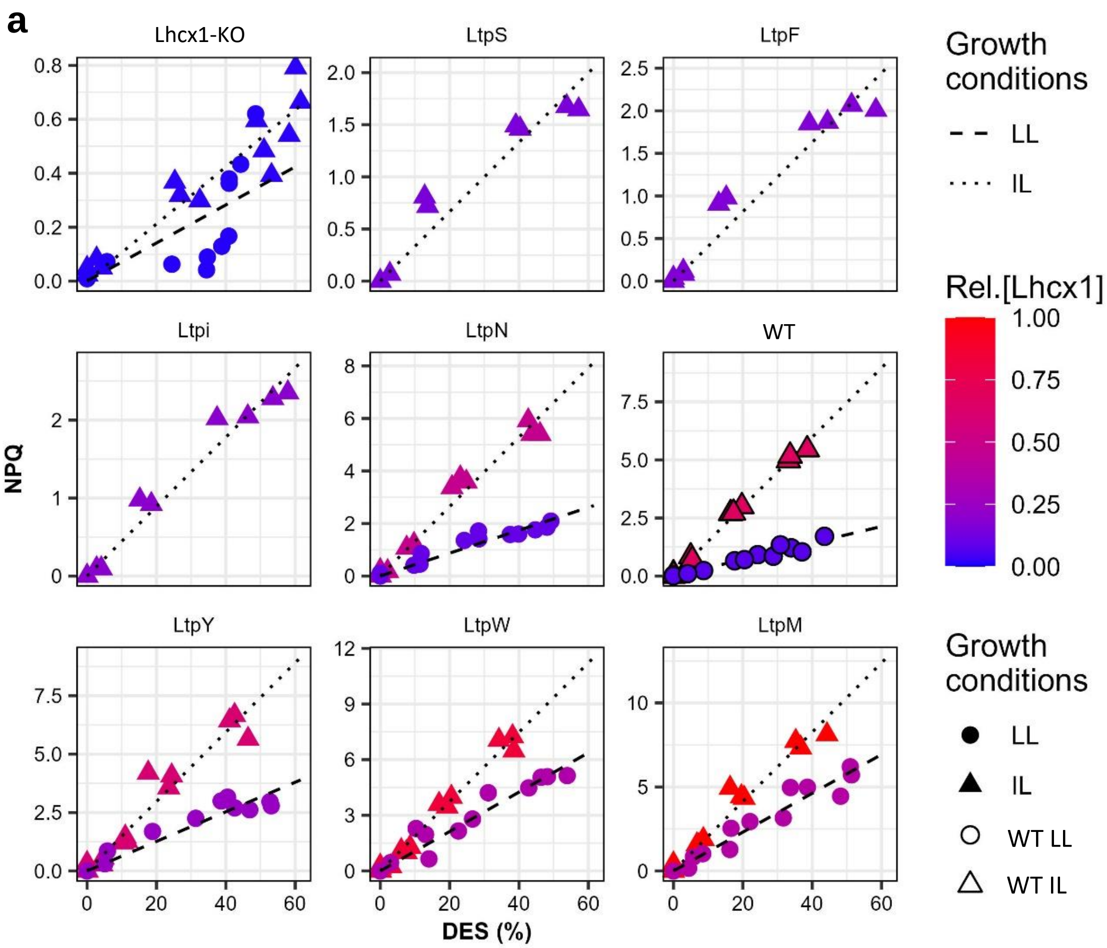

	Strains	Lhcx1	slope	SD	*n*
LL	KO	0.013	0.007	0.004	3
	WT	0.072	0.035	0.006	3
	LtpN	0.093	0.044	0.004	3
	LtpY	0.247	0.066	0.011	3
	LtpW	0.346	0.107	0.004	3
	LtpM	0.375	0.115	0.019	3
IL	KO	0.018	0.011	0.002	3
	LtpS	0.145	0.033	0.002	2
	LtpF	0.165	0.041	0.004	2
	Ltpi	0.198	0.045	0.003	2
	LtpN	0.476	0.132	0.010	3
	LtpY	0.606	0.150	0.021	3
	WT	0.676	0.150	0.005	3
	LtpW	0.842	0.188	0.019	3
	LtpM	1	0.208	0.012	3

Figure S4

b

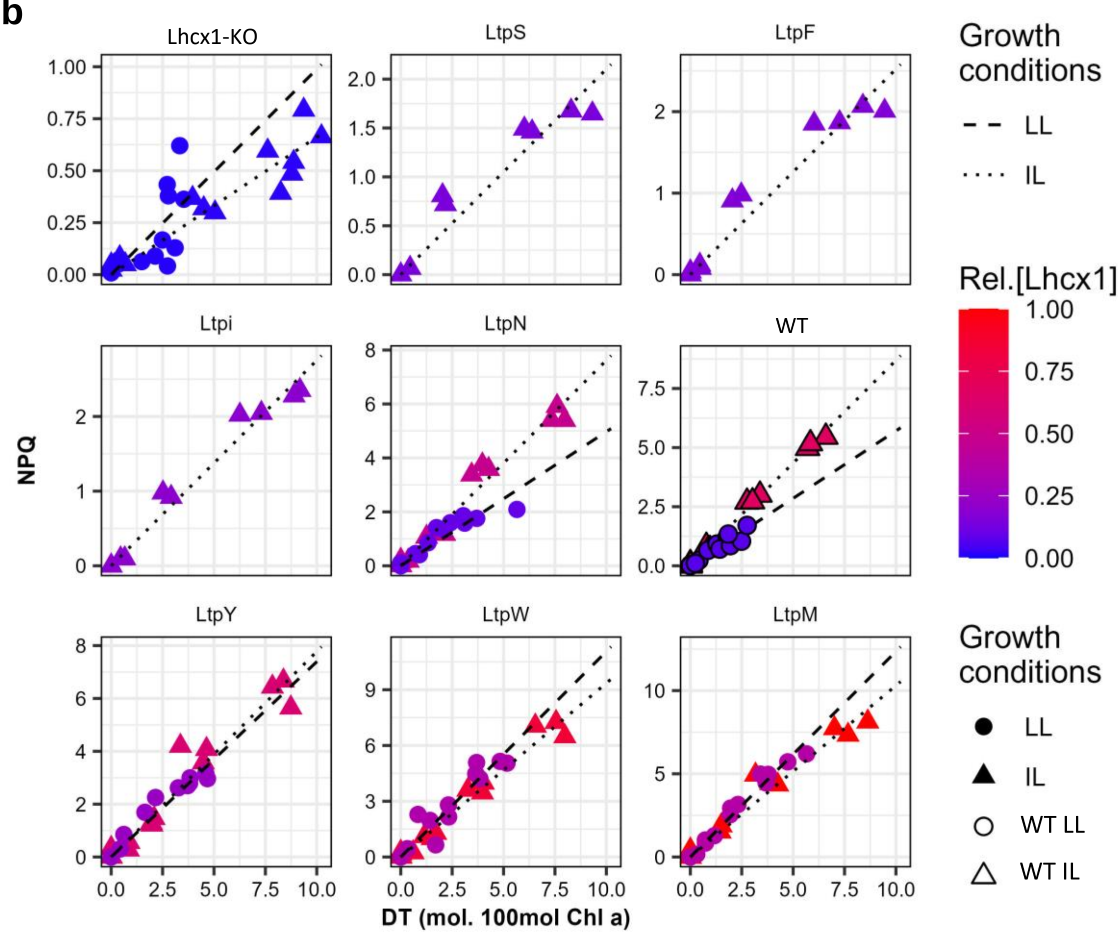

	Strains	Lhcx1	slope	SD	*n*
LL	Ko6	0.013	0.106	0.054	3
	Pt2	0.072	0.594	0.138	3
	LtpN	0.093	0.530	0.122	3
	LtpY	0.247	0.744	0.057	3
	LtpW	0.346	1.171	0.203	3
	LtpM	0.375	1.242	0.071	3
IL	Ko6	0.018	0.067	0.014	3
	LtpS	0.145	0.212	0.019	2
	LtpF	0.165	0.255	0.027	2
	Ltpi	0.198	0.276	0.011	2
	LtpN	0.476	0.764	0.046	3
	LtpY	0.606	0.786	0.101	3
	Pt2	0.676	0.872	0.027	3
	LtpW	0.842	0.952	0.131	3
	LtpM	1	1.035	0.067	3

Figure S4

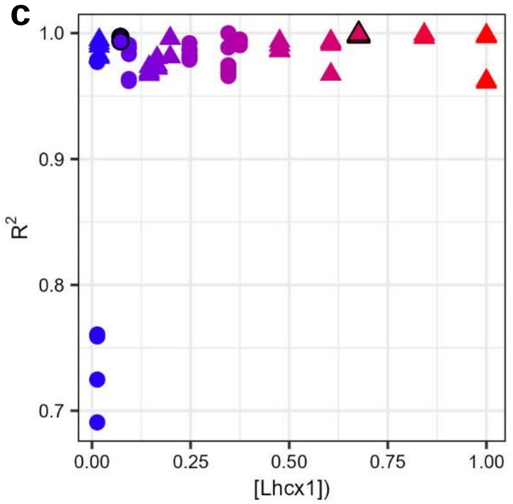

Fig. S4 | Slopes of the NPQ vs. DES or NPQ vs DT relationships, under steady-state light exposure, in all *Phaeodactylum tricornutum* strains.

All NPQ vs. DES (**a**) or NPQ vs DT (**b**) relationships measured in *P. tricornutum* wildtype (WT) and all Lhcx1-mutants strains under increasing light intensities at steady-state, following cultivation under low light (LL, circles) and intermittent light (IL, triangles) growth conditions. All individual experiments (comprising 5 data points) were fitted linearly and all obtained R^2 are plotted as a function of [Lhcx1] (**c**), with R^2>0.95 in all fits independently of Lhcx1 concentration, except in Lhcx1-KO**.** For all species*growth condition, the mean slope and are calculated shown under the plots in (**a**) and (**b**). These mean values of NPQ/DES and NPQ/DT slopes are used for the linear regressions vs. Lhcx1 concentration in Table 1 of the main manuscript.

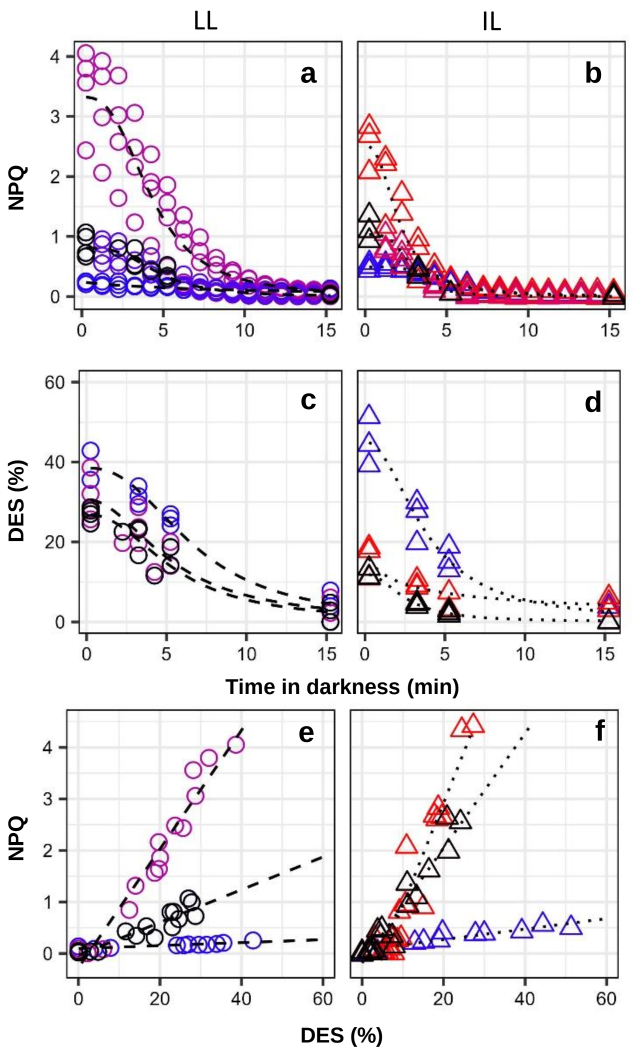

Fig. S5 | Relaxation of NPQ and DES in darkness in *Phaeodactylum tricornutum* wildtype and Lhcx1 mutants.

NPQ (**a, b**) and de-epoxidation state (DES) (**c, d**) relaxation kinetics in *P. tricornutum* wildtype (WT, black outline symbols), Lhcx1-KO (blue symbols) and the Lhcx1 overexpressor strain, LtpM (pink and red symbols), in the dark, after NPQ induction under 6 min of high light (450 µmol photons m^{-2} s^{-1}). Data for cells grown under low light (LL, open circles) (**a**, **c**) and intermittent light (IL, open triangles) (**b**, **d**) are shown. Based on the first 4 panels, the NPQ vs. DES linear relationships were plotted for LL (**e**) and IL (**f**). Relationships between NPQ. DES and diatoxanthin, in all replicates of all strains*growth conditions are shown in Fig. S6.

Figure S6

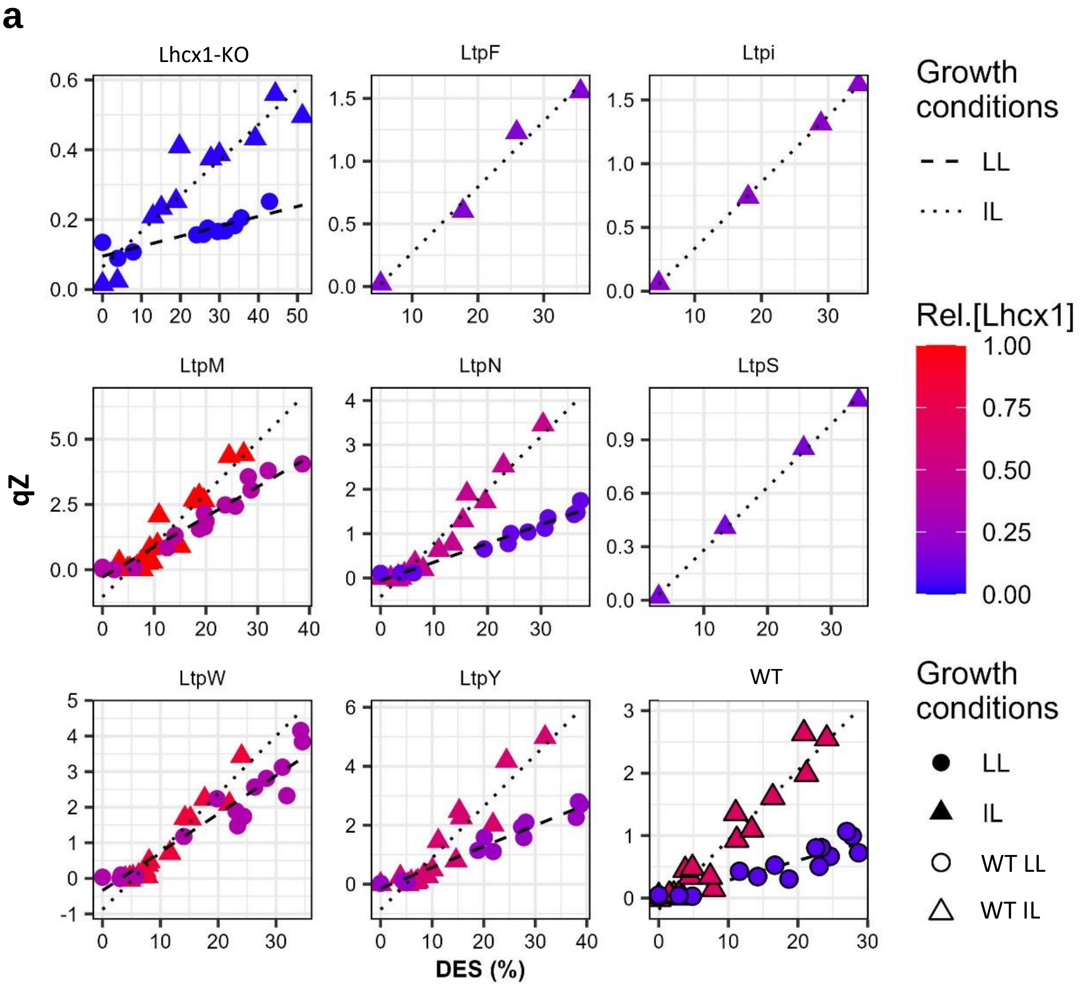

	Strains	Lhcx1	slope	SD	y0	SD	*n*
LL	Lhcx1-KO	0.013	0.005	0.000	0.043	0.020	3
	WT	0.072	0.031	0.007	-0.013	0.039	4
	LtpN	0.093	0.041	0.004	-0.032	0.053	3
	LtpY	0.247	0.069	0.007	-0.074	0.067	3
	LtpW	0.346	0.100	0.021	-0.187	0.144	4
	LtpM	0.375	0.110	0.013	-0.154	0.164	4
IL	Lhcx1-KO	0.018	0.011	0.002	0.030	0.023	3
	LtpS	0.145	0.034		-0.038		1
	LtpF	0.165	0.047		-0.119		1
	Ltpi	0.198	0.048		-0.087		1
	LtpN	0.476	0.106	0.021	-0.237	0.094	4
	LtpY	0.606	0.150	0.044	-0.477	0.412	4
	WT	0.676	0.106	0.021	-0.130	0.118	5
	LtpW	0.842	0.129	0.022	-0.451	0.136	4
	LtpM	1.000	0.175	0.021	-0.639	0.274	5

Figure S6

b

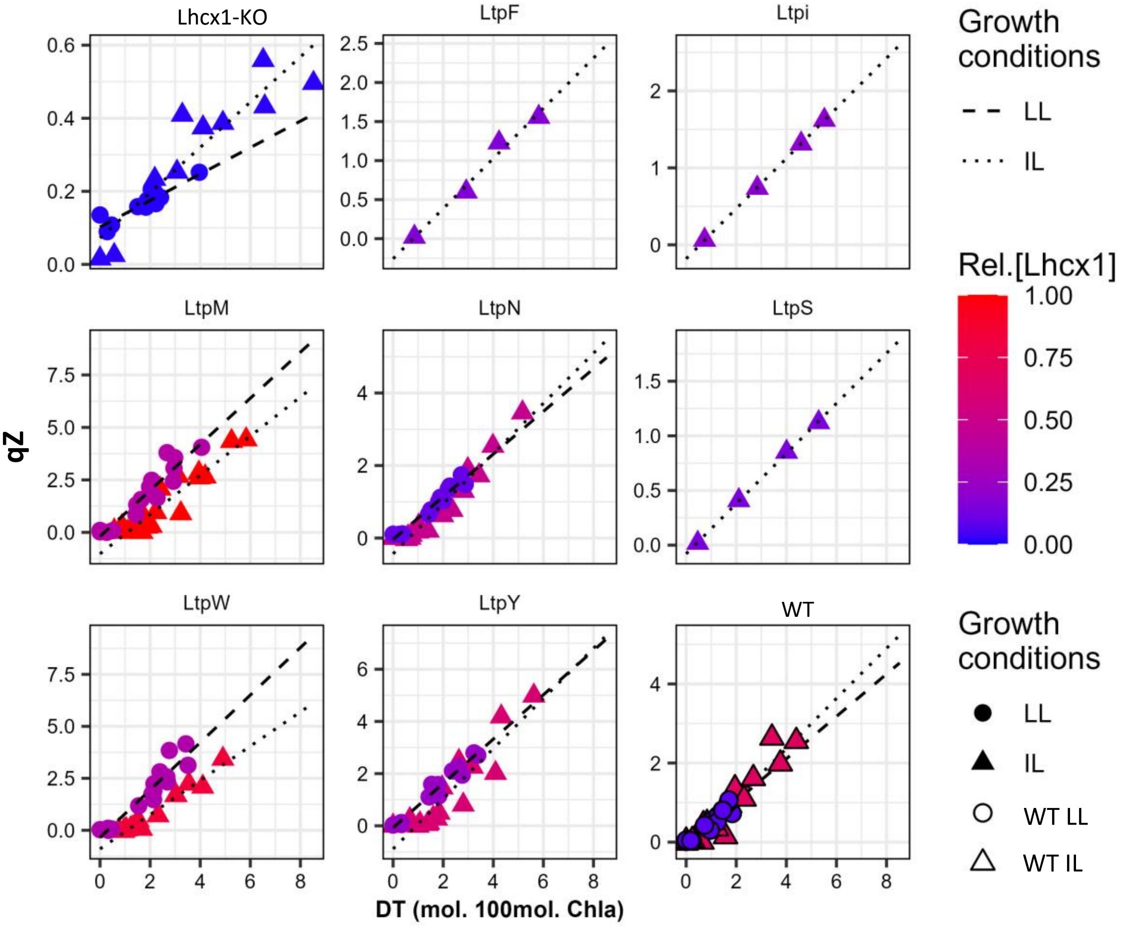

	Strains	Lhcx1	slope	SD	y0	SD	*n*
LL	Lhcx1-KO	0.013	0.065	0.018	0.045	0.023	3
	WT	0.072	0.515	0.093	-0.011	0.041	4
	LtpN	0.093	0.586	0.082	-0.030	0.059	3
	LtpY	0.247	0.856	0.087	-0.063	0.058	3
	LtpW	0.346	1.057	0.226	-0.180	0.140	4
	LtpM	0.375	1.102	0.255	-0.151	0.171	4
IL	Lhcx1-KO	0.018	0.072	0.015	0.031	0.024	3
	LtpS	0.145	0.220		-0.042		1
	LtpF	0.165	0.290		-0.119		1
	Ltpi	0.198	0.304		-0.085		1
	LtpN	0.476	0.593	0.116	-0.235	0.106	4
	LtpY	0.606	0.802	0.220	-0.467	0.400	4
	WT	0.676	0.625	0.116	-0.135	0.119	5
	LtpW	0.842	0.640	0.082	-0.446	0.131	4
	LtpM	1.000	0.853	0.078	-0.671	0.269	5

Figure S6

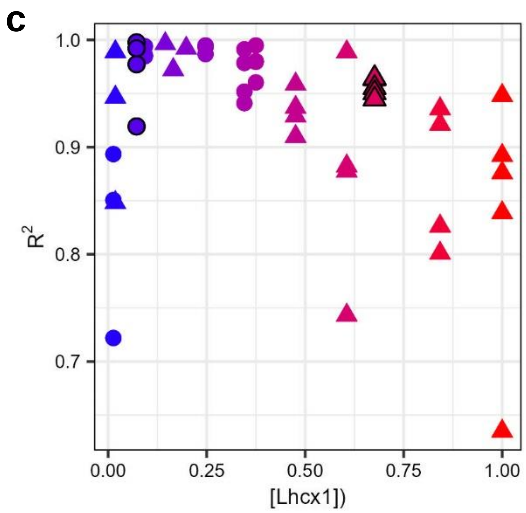

Fig. S6 | Slopes of the qZ component of NPQ vs. DES or, vs. DT relationships, during relaxation in darkness, in all *Phaeodactylum tricornutum* strains.

All relationships between the rapidly reversible, diatoxanthin-dependent, qZ component of NPQ vs. DES (**a**) or vs. DT (**b**) measured in *P. tricornutum* wildtype (WT) and all Lhcx1-mutants over different time points of qZ relaxation in darkness, following cultivation under low light (LL, circles) and intermittent light (IL, triangles) growth conditions. All individual experiments (comprising time=0 plus 4 time points of relaxation) were fitted linearly and all obtained R^2 are plotted as a function of [Lhcx1] in (**c**). For IL strains, there was a deviation from proportionality between qZ and DES or DT, leading to lower R^2 when fitted with a linear function (see Discussion). For all species*growth condition, the mean slope and SD are calculated and shown under the plots in (**a**) and (**b**). These mean values of qZ/DES and qZ/DT slopes are used for the linear regressions vs. Lhcx1 concentration in Table 1 of the main manuscript.

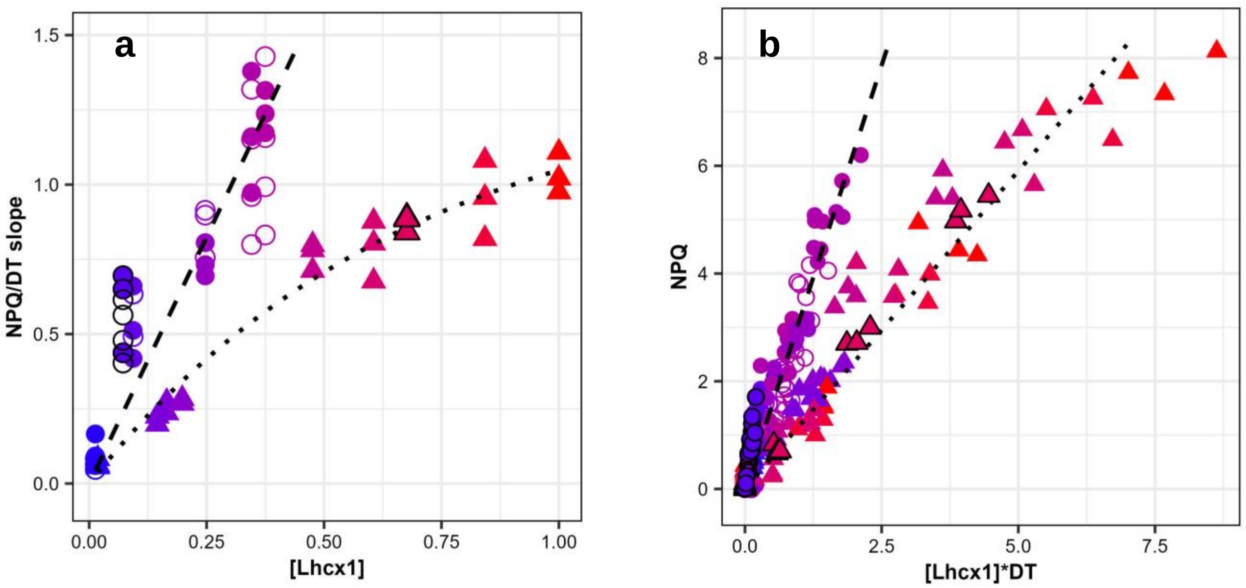

Fig. S7 | Relationships between NPQ, DT and Lhcx1 in all *Phaeodactylum tricornutum* strains.

The slopes of the NPQ/DT relationships measured under different light intensities at steady-state (closed symbols) and during NPQ relaxation in darkness (open symbols) are plotted against the relative Lhcx1 concentration (**a**). The panel **a** combines different *P. tricornutum* strains (black outline symbols represent wildtype) acclimated to low light (LL, circles) or intermittent light (IL, triangles). All 291 NPQ data points corresponding to the experiments from which the slopes in (**a**) were calculated are plotted vs. DT×[Lhcx1] (**b**).

Figure S8

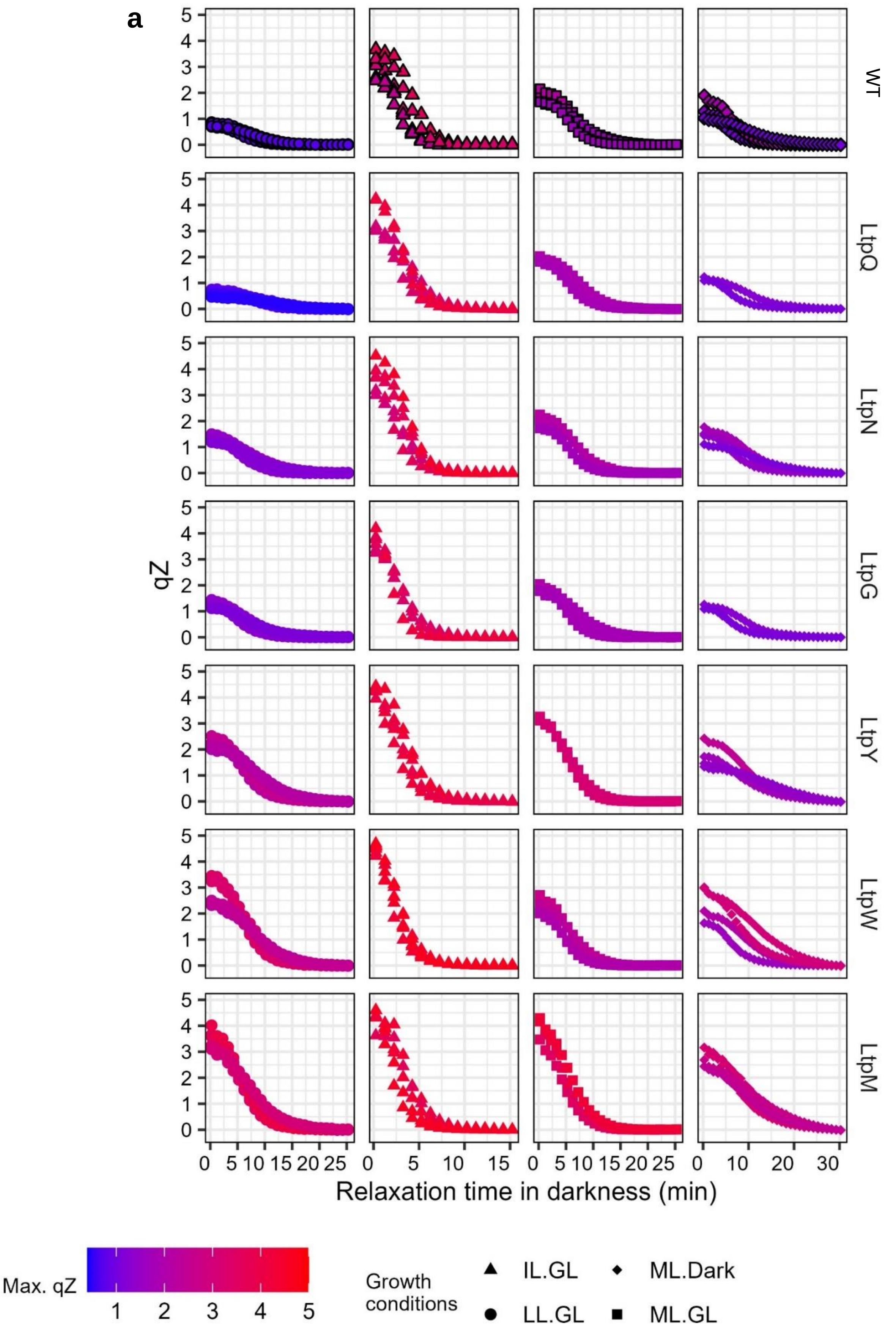

a
LL
GL
IL
GL
ML
GL
ML
Dark
WT
LtpQ
LtpN
LtpG
LtpY
LtpW
LtpM
qZ
Relaxation time in darkness (min)
Max. qZ
Growth conditions
IL.GL
ML.Dark
LL.GL
ML.GL

Figure S8

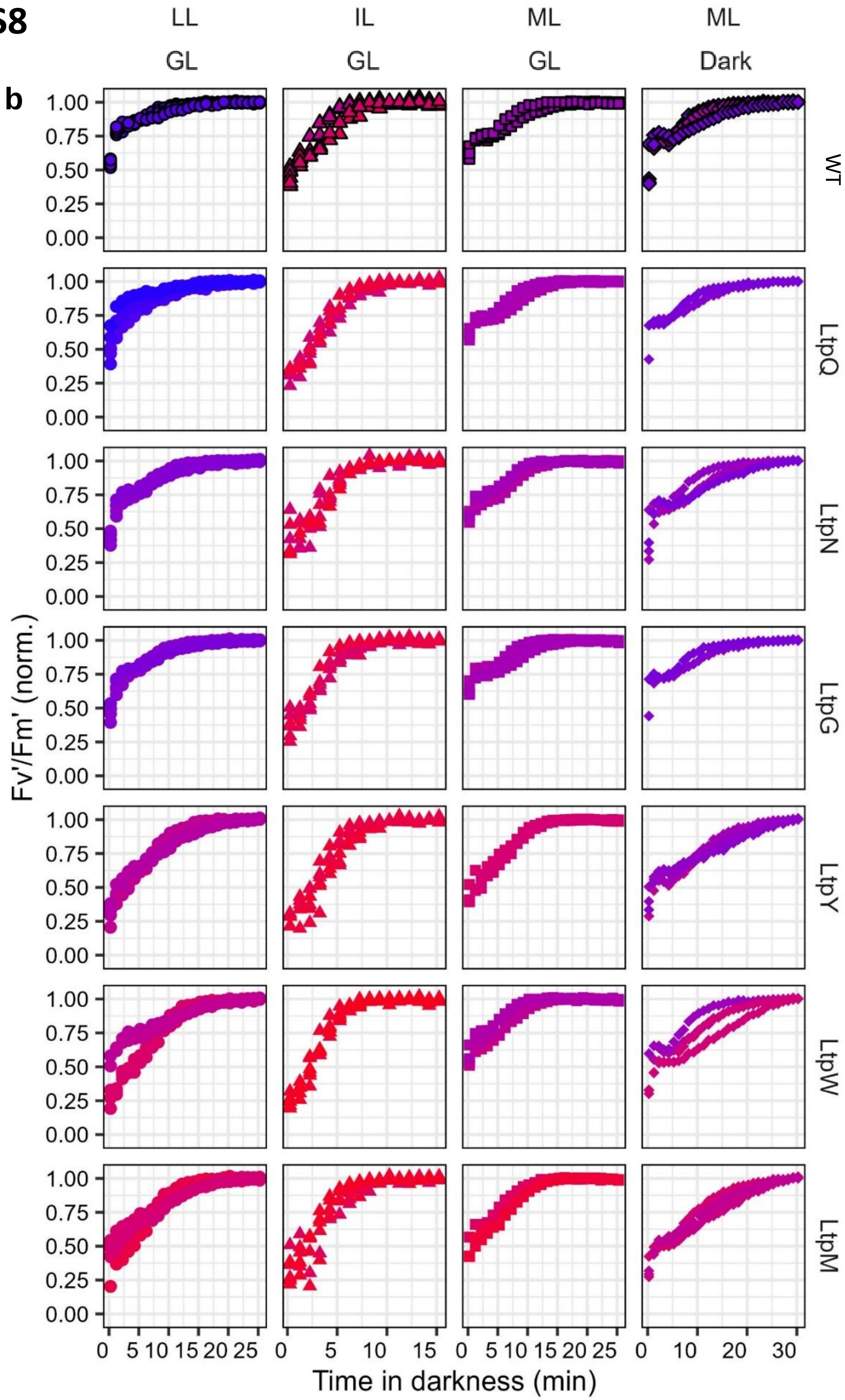

LL
GL
IL
GL
ML
GL
ML
Dark
b
1.00
0.75
0.50
0.25
0.00
WT
LtpQ
LtpN
LtpG
LtpY
LtpW
LtpM
Fv'/Fm' (norm.)
0 5 10 15 20 25
0 5 10 15
0 5 10 15 20 25
0 10 20 30
Time in darkness (min)

Figure S8

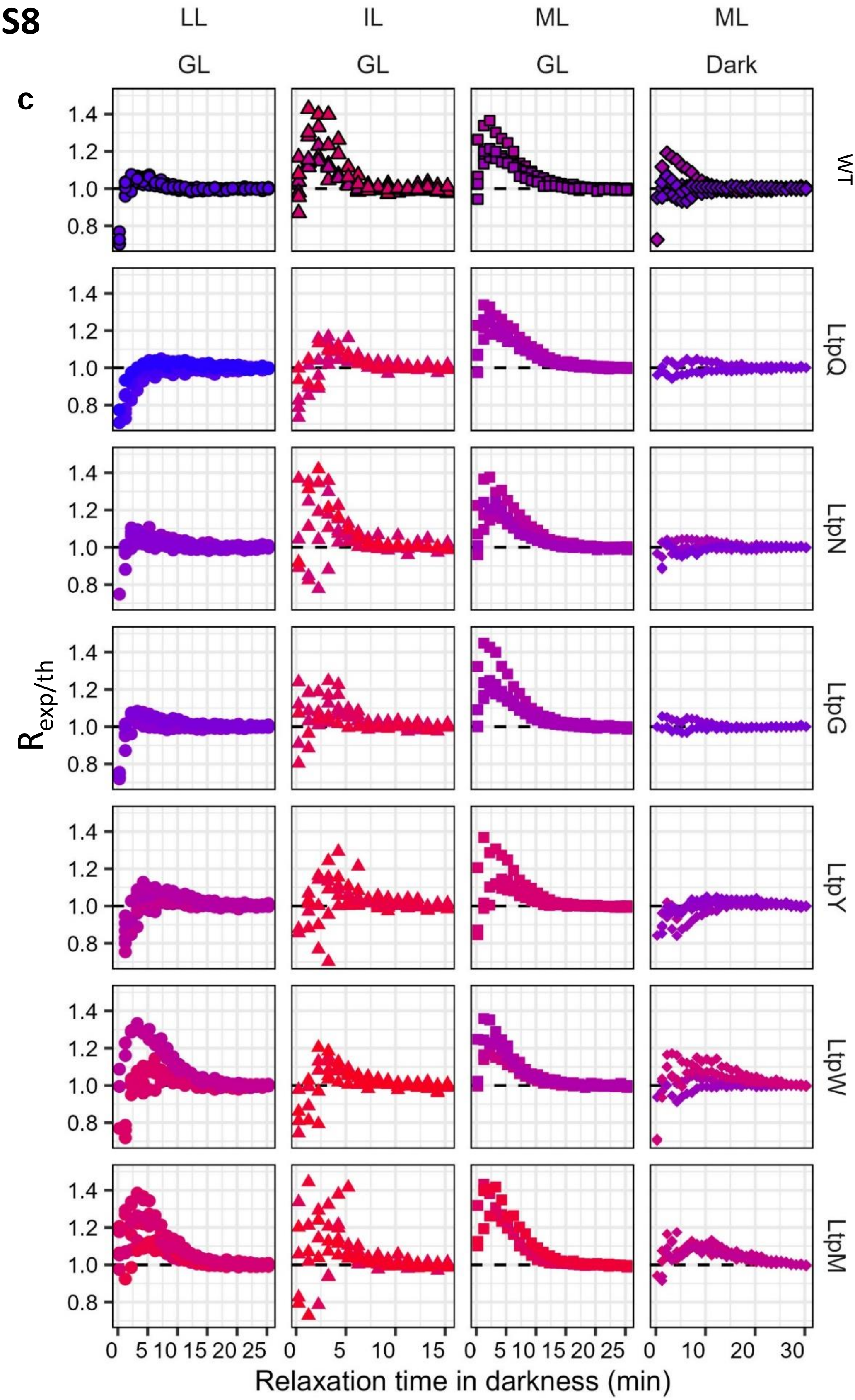

c
LL
GL
IL
GL
ML
GL
ML
Dark
WT
LtpQ
LtpN
LtpG
LtpY
LtpW
LtpM
R exp/th
1.4
1.2
1.0
0.8
0 5 10 15 20 25
0 5 10 15
0 5 10 15 20 25
0 10 20 30
Relaxation time in darkness (min)

Fig. S8 | Time dependence of the qZ component of NPQ, F_V'/F_M' and the Ratio of experimental-to-theoretical F_V'/F_M' ($R_{exp/th}$) during relaxation in darkness, in all *Phaeodactylum tricornutum* strains.

For all strains and growth conditions (low light (LL), intermittent light (IL), moderate light (ML) under growth light (GL) or after 40h of dark acclimation (Dark)), the kinetics of the rapidly reversible, diatoxanthin-dependent, qZ component of NPQ relaxation (**a**), F_V'/F_M' normalized to its fully relaxed value (**b**), and $R_{exp/th}$ (**c**) were measured after qZ induction under high light (6 min, 600 (LL, IL) or 750 µmol photons $m^{-2}\ s^{-1}$ (ML)). Raw data for LL growth conditions were used to derive similar parameters published in [1] and are shown here after reanalysis in the context of testing the "SV-lake" model.

Figure S9

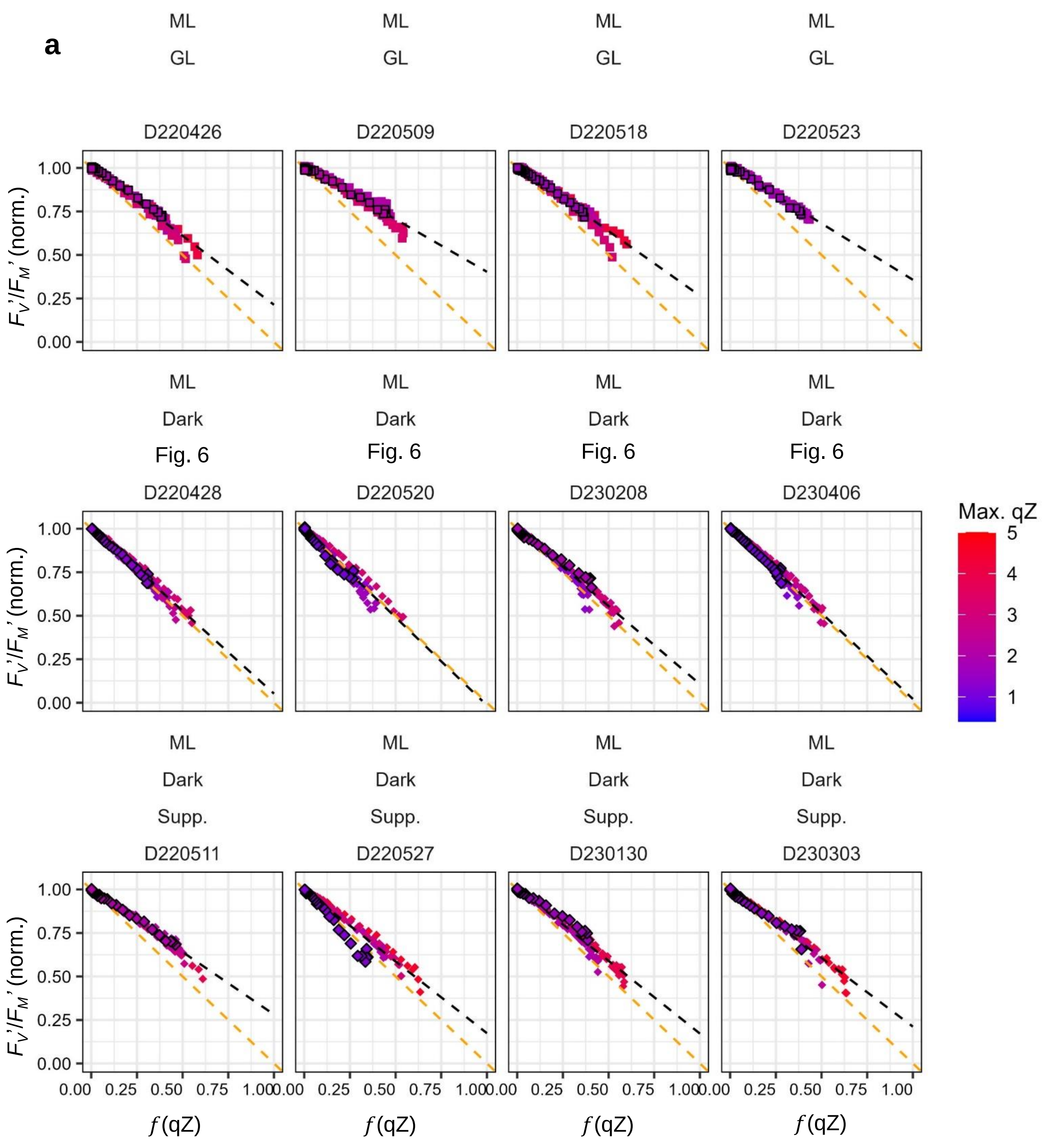

a
ML
GL
D220426
D220509
D220518
D220523
ML
Dark
Fig. 6
D220428
D220520
D230208
D230406
ML
Dark
Supp.
D220511
D220527
D230130
D230303
F_V'/F_M' (norm.)
f(qZ)
Max. qZ
0.00
0.25
0.50
0.75
1.00
1
2
3
4
5

Figure S9

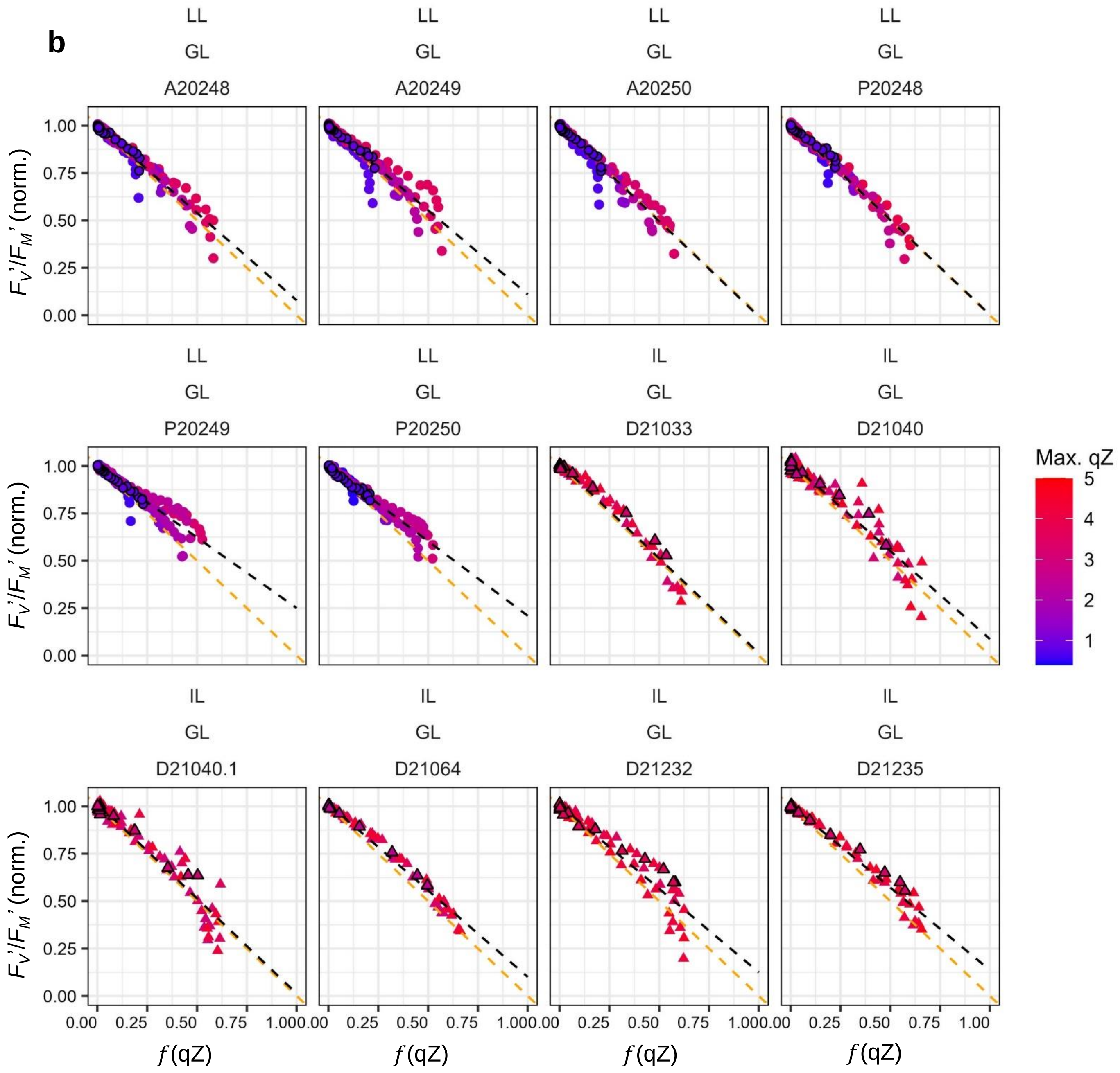

b
LL
GL
A20248
LL
GL
A20249
LL
GL
A20250
LL
GL
P20248
LL
GL
P20249
LL
GL
P20250
IL
GL
D21033
IL
GL
D21040
IL
GL
D21040.1
IL
GL
D21064
IL
GL
D21232
IL
GL
D21235
F_V'/F_M' (norm.)
f(qZ)
Max. qZ
0.00
0.25
0.50
0.75
1.00
1
2
3
4
5

Figure S9

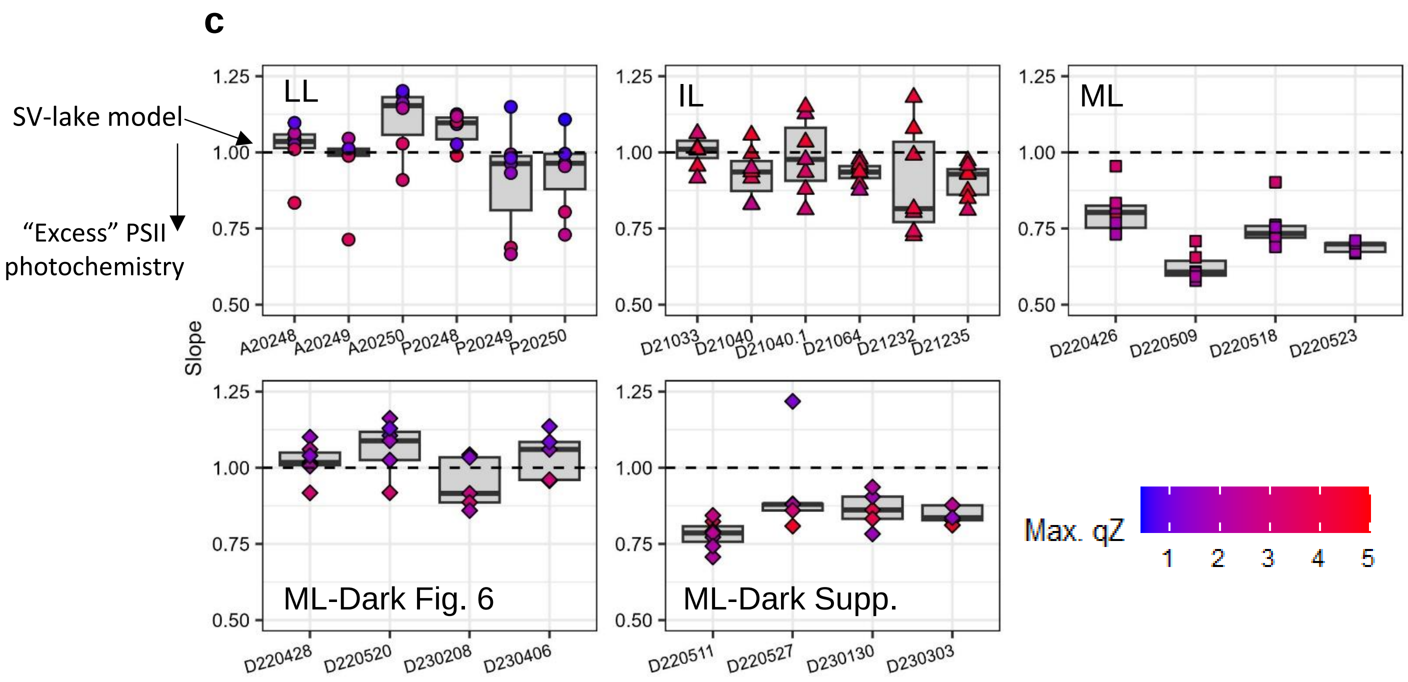

Fig. S9 | Normalized F_V'/F_M' vs. its relative decrease due to qZ (f(qZ)), in all *Phaeodactylum tricornutum* strains, for all experiments/days of measurements and growth conditions.

Every experiment, spanning 5 to 7 strains (including wildtype (black outline symbols)) cultivated under a same growth condition, for which the relationship between F_V'/F_M' and f(qZ) was tested is shown, with the dashed black line representing linear regression analysis ran on all strains together, compared to the theoretical slope of -1 (orange dashed lines). In (**a**) strains are acclimated to moderate light (ML), sampled directly under growth light (GL, squares) or after 40 h of "Dark" acclimation (diamonds), for which we distinguish experiments of near exact agreement with the Stern-Volmer (SV)-lake model theory shown in Fig. 6 and days of experiment where Dark treatment improved agreement with theory compared to ML-GL, but not fully. In (**b**) strains are acclimated to low light (LL, circles) and intermittent light (IL, triangles). The absolute value of the slope of linear regression ran on every individual strain for a given day of experiment and growth condition are shown in (**c**). Absolute slope value of 1 are expected by the SV-lake model, while absolute slope values below 1 represent "excess" of PSII photochemistry (see main manuscript).

Figure S10

a

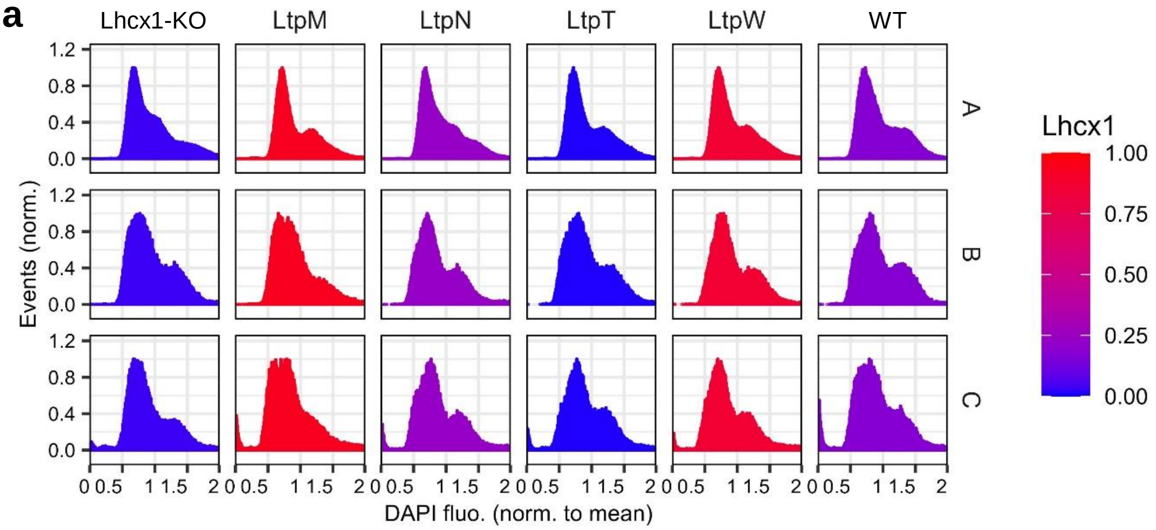

b

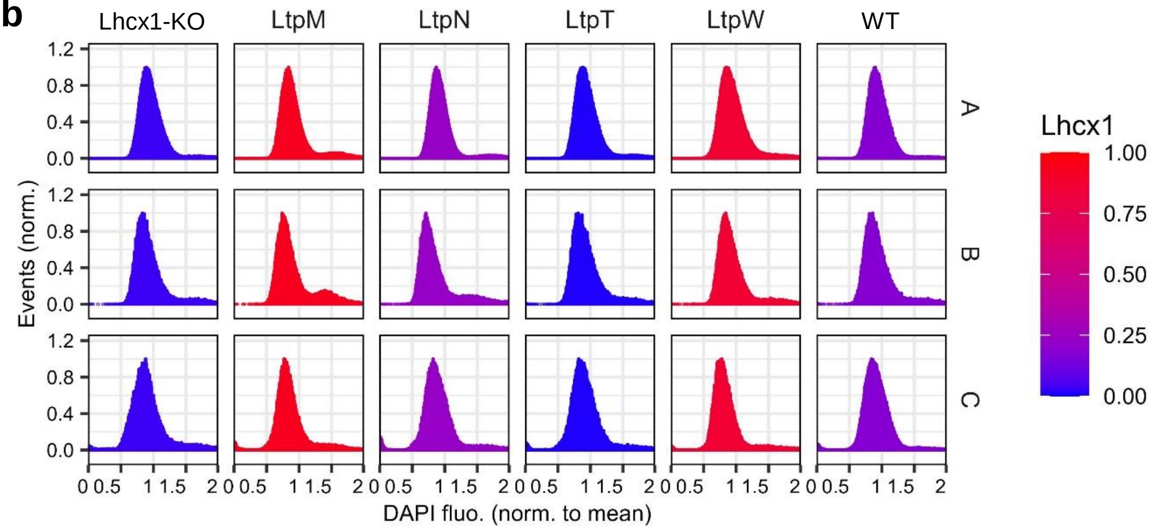

c

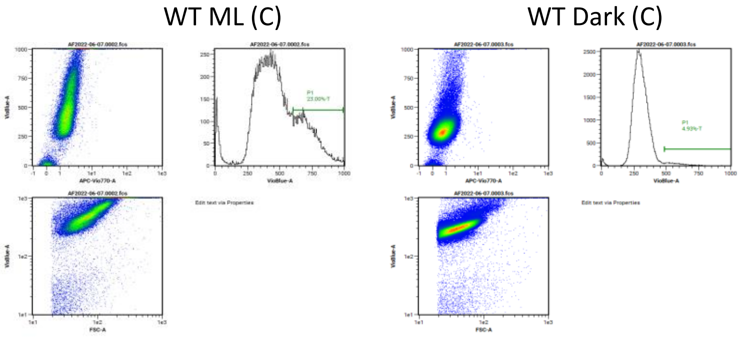

d

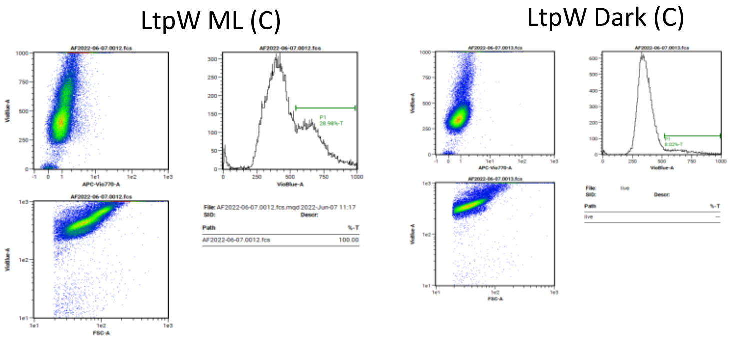

Figure S10

e

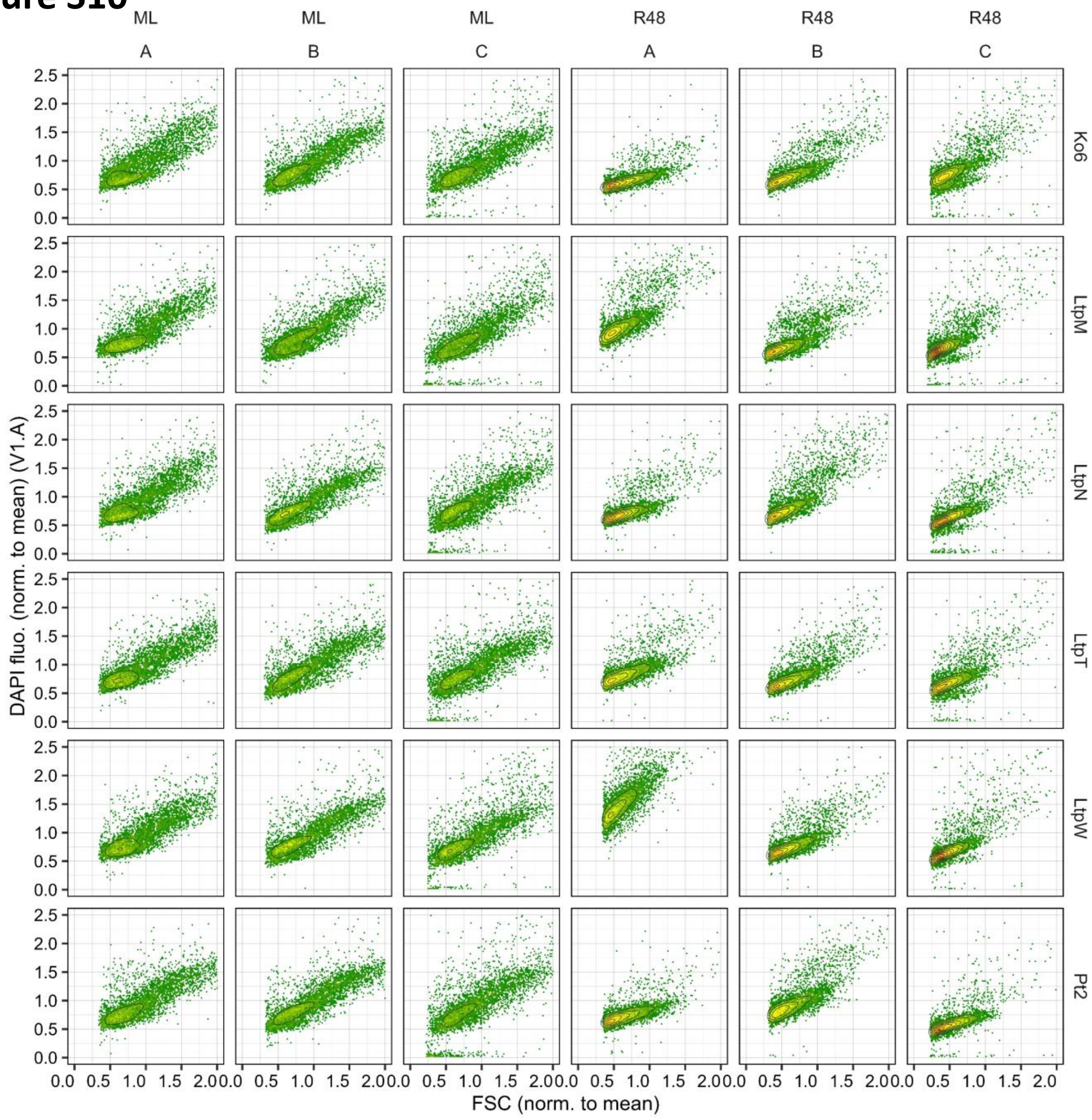

Fig. S10 | Assessing physiological heterogeneity with and without dark acclimation in *Phaeodactylum tricornutum* wildtype and different Lhcx1 mutants.

Cytograms showing events (≥ 50, 000 recorded) of DAPI-fluorescence (V1.A channel), a proxy of DNA content per cell, in cultures of different *P. tricornutum* strains grown under moderate light (ML) and sampled either directly under growth light (**a**) or after 40 h of "Dark" acclimation, before centrifugation and fixation in cold EtOH (**b**). This protocol commonly used to synchronize cells' physiology (see Methods and [2]) served as the basis to explore the "heterogeneous Stern-Volmer hypothesis" in Text S2. Examples of MACSquantify software (version 3.0.1) output and gating on V1.A histogram to approximate the proportion of each population of wildtype (Replicate C) (**c**) and LtpW (Replicate C) for ML and Dark treatment (**d**). DAPI fluorescence vs. forward scatter (FSC) in all replicates (strain*Treatment), with the mean of both parameters being calculated over all events recorded for ML and Dark treatments, plot made with R software and binning set at 100 (**e**).

References

1: Giovagnetti, V. Jaubert, M, Shukla MK, Ungerer P, Bouly JP, Falciatore A, and Ruban AV. Biochemical and molecular properties of LHCX1, the essential regulator of dynamic photoprotection in diatoms. *Plant Physiology* **188**, 509–525 (2022).

2: Huysman MJ, Martens C, Vandepoele K, Gillard J, Rayko E, Heijde M, Bowler C, Inzé D, Peer Y, De Veylder L, *et al.* 2010. Genome-wide analysis of the diatom cell cycle unveils a novel type of cyclins involved in environmental signaling. *Genome Biology* **11**: R17.

Supplementary Text S1 | Mathematical and theoretical foundations of the “SV-lake” model.

In this Appendix, we outline the fundamental principles of the “lake” model and explain how photosynthetic parameters can be derived from chlorophyll *a* fluorescence measurements within this theoretical framework. Additionally, we expend this model to the situation where the regulated heat dissipation process (reversible non-photochemical quenching) is entirely attributed to a quencher Q of concentration [Q], and refer to this expanded model as the “SV-lake” model.

Within the “lake” model, the photosynthetic unit includes numerous photosystem II complexes embedded in a shared light-harvesting antenna, all competing for excitons [1,2]. The yield of each process involved in excited chlorophyll relaxation is then determined by the kinetic competition between these processes. The yields of fluorescence (Φ_F), intrinsic losses (sum of fluorescence and non-regulated heat, Φ_{NO}), photochemistry (Φ_{PSII}) and non-photochemical quenching (Φ_{NPQ}) are described by the following equations:

**Equation 5**	$\Phi_F = k_F / (k_F + k_H + k_p \times [Q_A] + \kappa_{NPQ})$
**Equation 6**	$\Phi_{NO} = (k_F + k_H) / (k_F + k_H + k_p \times [Q_A] + \kappa_{NPQ})$
**Equation 7**	$\Phi_{PSII} = k_p \times [Q_A] / (k_F + k_H + k_p \times [Q_A] + \kappa_{NPQ})$
**Equation 8**	$\Phi_{NPQ} = \kappa_{NPQ} / (k_F + k_H + k_p \times [Q_A] + \kappa_{NPQ})$

Here, k_F, k_H, k_p and κ_{NPQ} represent the rate constants for radiative and non radiative dissipation, photochemical and non-photochemical quenching, respectively. The term $[Q_A]$ denotes the concentration of open reaction centers with oxidized Q_A.

Within this theoretical framework, fluorescence yield can provide valuable information about the photochemical and non-photochemical yields, provided that PSI fluorescence – considered minor and of constant yield- is not considered [3]. The fluorescence measured from a photosynthetic sample in a given instrument can be expressed as the product of a factor α (describing the instrument and sample, e.g., the geometry of the instrument, the percentage of fluorescence detected, number of photosynthetic units in the sample), the fluorescence yield (Φ_F) and the light irradiance E.

**Equation 9**	$F = \alpha \times E \times k_F / (k_F + k_H + k_p[Q_A] + \kappa_{NPQ})$

From there, the fluorescence level can be derived in different situations: F_0 and F_m represent the fluorescence levels when all PSIIs are open ($[Q_A] = 1$) or closed (($[Q_A] = 0$) in the absence of NPQ, respectively. Whereas F_0' and F_m' are their counterparts when NPQ is present.

**Equation 10**	$F_0 = \alpha \times E \times k_F / (k_F + k_H + kp)$

**Equation 11**<br>**Equation 12**<br>**Equation 13**	$F_m = \alpha \times E \times k_F / (k_F + k_H)$<br>$F_0' = \alpha \times E \times k_F / (k_F + k_H + kp + \kappa_{NPQ})$<br>$F_m' = \alpha \times E \times k_F / (k_F + k_H + \kappa_{NPQ})$

By combining equations 6, 7, 8, 9, 10 and 13, we find that that the yields of intrinsic losses, photochemistry or of non-photochemical quenching can be derived from such fluorescence measurements.

**Equation 14**<br>**Equation 15**<br>**Equation 16**	$\Phi_{NO} = F/ F_m$<br>$\Phi_{PSII} = (F_m' – F) / F_m'$<br>$\Phi_{NPQ} = F / F_m' - F / F_m$

We can also calculate the maximum (F_v/F_m) or potential (F_v'/F_m') (when NPQ remains present after dark acclimation allowing to re-oxidize Q_A) quantum yield of PSII for photochemistry by combining equations 10 to 13:

**Equation 17**<br>**Equation 18**	$F_v/F_m = (F_m – F_0)/F_m) = k_p / (k_F + k_H + k_p)$<br>$F_v'/F_m' = (F_m' – F_0')/F_m') = k_p / (k_F + k_H + k_p + \kappa_{NPQ})$

For a fluorophore in a homogeneous solution, the extent of fluorescence quenching by a quencher Q is expressed as the product of the quencher concentration and its quenching rate coefficient. The Stern-Volmer equation postulates a kinetic competition between radiative relaxation (fluorescence) and relaxation of the excited fluorophore by Q and expresses the NPQ as the product of the Stern-Volmer (SV) quencher Q concentration and its quenching coefficient k_{SV}. For the photosynthetic unit, we call this the “SV-lake” model, the theoretical framework where NPQ is entirely due to changing concentration of a non-photochemical quencher Q, behaving as a SV quencher:

**Equation 19**	$\kappa_{NPQ} = k_{SV} \times [Q]$

Combining equations 16 and 19 gives the equation 1 displayed in the main text:

**Equation 1**	$NPQ = F_m/ F_m' – 1 = k_{NPQ}/(k_F + k_H) \times [Q] = k_{SV} [Q]$

In a “SV-lake” model, the product of the SV quencher concentration and its quenching coefficient can therefore be assessed through the sole measurements of fluorescence levels at closed reaction centers (F_m and F_m').

In the “lake” or “SV-lake” models, every excited chlorophyll has the same probability of encountering a photochemical or non-photochemical quencher and the yield of both processes is –again- given by the kinetic competition between them. Therefore, it is possible to determine the influence of NPQ on photochemistry. The influence of NPQ on the fluorescence level at open reaction center (F_0’, maximal yield for photochemistry) can be obtained by combining equations 10, 12 and 10:

**Equation 20**	$F_0/ F_0' - 1 = k_{NPQ}/(k_F + k_H + k_p) \times [Q] = k_{SV0} [Q]$

Combining equations 1 and 20 gives equation 21 which expresses the change in the efficiency of the SV quencher due to photochemistry:

**Equation 21**	$k_{SV0} / k_{SV} = F_0/ F_m$

Another way to express the interplay between photochemical and non-photochemical quenching is to calculate the influence of NPQ on the potential yield of PSII for photochemistry (F_v'/F_m'). Combining 15, 16 and 17, which gives equation 22:

**Equation 22**	$F_v'/F_m' = (F_v/F_m) / (1 + (1 - F_v/F_m) \times NPQ)$

We can define $f(NPQ)$ as the expected relative decrease of potential quantum yield of PSII (F_v'/F_m') for photochemistry due to NPQ (using the maximum quantum yield F_v/F_m as a reference):

**Equation 23**	$f(NPQ) = (F_v/F_m - F_v'/F_m')/(F_v/F_m)$<br>$= (1 - F_v/F_m) \times NPQ/(1 + (1 - F_v/F_m) \times NPQ)$

The combination of equations 22 and 23 provides the equation 2 from the main text, which we preferred to the equation 19 because it allows to confront our experimental data (relationship between F_v'/F_m' and $f(NPQ)$) with a theoretical relationship which is linear. It is important to note that testing the validity of the “SV-lake” model through equation 23 (our work) is absolutely equivalent to testing it through equation 22 (as in [4]) or through equation 21 [5]. In another study [6], the evolution of functional absorption cross-section (σPSII) was studied as a function of NPQ. Since the functional cross-section (σPSII) is the

product of the optical cross-section of PSII (optical-σPSII) and the potential yield of PSII for photochemistry (F_v'/F_m') when NPQ represents only heat-dissipation processes in the antenna[7,8], the same NPQ-dependencies are expected for F_v'/F_m' or for σPSII.

Supplementary Text S2 | Empirical exploration of the "heterogeneous Stern-Volmer (SV) lake" model.

In this section, we want to test the "heterogeneity hypothesis" (see Main text). We aim to demonstrate that the observed "excess" of PSII photochemistry (Fig. 4 and 6), relative to that expected for a given NPQ/qZ level in the homogeneous SV-lake model, is consistent with theoretical expectations if different PSII "lakes" with varying properties exist (e.g., between cells). These varying properties may include different concentrations of photochemical or non-photochemical quenchers ([Q_A] or [Q], respectively) or variations in the rate constants of the different dissipation pathways (photochemistry (k_P), fluorescence (k_F), intrinsic heat losses (k_H), and SV-NPQ (k_{SV}), see Supplementary Text S1).

To explore this, we performed simulations incorporating cell heterogeneity, representing two scenarios: two cell subpopulations (60% and 40%) under moderate light (ML), and two subpopulations (95% and 5%) after 40h dark acclimation. These stoichiometries were chosen on the basis of the distribution of DNA content per cell in our samples, based on DAPI-fluorescence (Fig. S9). The smaller subpopulation (population 2) had a higher DNA content per cell and biovolume than the larger one (population 1), indicating cells in the division cycle [9]. Based on single-cell fluorescence data from[10], *Pseudo-nitzschia multistriata* cells undergoing sexual reproduction have ≈40% lower F_V/F_M and higher NPQ. Accordingly, F_V/F_M values of 0.7 and 0.4 and maximum NPQ/qZ values of 2 and 5 were assigned to populations 1 and 2, respectively.

Simulations followed the evolution of qZ and the potential quantum yield of PSII (F_V'/F_M') of populations 1 and 2, during dark relaxation. We considered that qZ decayed monoexponentially from its maximal value with a half-time of 4 min, and the evolution of F_V'/F_M' was calculated based on the expected relationship between F_V'/F_M' and qZ with Q_A always fully oxidized (as expected during dark relaxation, see Text S1). In the simulations below (left: population 1; middle: population 2), those relaxations are shown (top panels), as well as the Ratio of experimental-to-theoretical F_V'/F_M' ($R_{exp/th}$, middle panels) and the F_V'/F_M' (normalized to its maximum value) vs. f(qZ) plot (bottom panels). In both populations, $R_{exp/th}$ was, as expected, consistently equal to 1 and F_V'/F_M' vs. f(qZ) adhered to the expected relationship.

Using these results, we reconstructed F_0' and F_M' kinetics for both populations 1 and 2, assuming an initial F_M = 1 (see Table at the bottom of each simulation). Combining these subpopulations with stoichiometries reflecting ML (60% and 40%) and 40h dark acclimation (95% and 5%), we predicted the relaxation behaviour of heterogeneous samples (see Table below the simulations). The simulations reveal (right columns):

- **ML sample**: a $R_{exp/th}$ significantly greater than 1 (1.3 at the onset of relaxation), with a notable deviation from the theoretical slope -1 in the F_V'/F_M' vs. f(qZ) plot (orange dotted line). These results indicate a significant apparent deviation from the SV-lake model.
- **40 h dark acclimation sample**: $R_{exp/th}$ remained close to 1 (maximum value 1.03), with minimal deviation from the expected F_V'/F_M' vs. f(qZ) relationship.

For both samples, the best linear fit of F_V'/F_M' vs. f(qZ) (dashed green line) closely matches the experimental data (see Figs. 4d and 6d, where the dashed green lines from these simulations are shown). These results support the "heterogeneity hypothesis" described in the main text: apparent deviations from the SV-lake model appear when heterogeneity is taken into account, *even though individual subpopulations conform to the SV-lake model*.

Text S2: Scenario of 60/40% populations, typical of ML acclimation (Fig. 4)

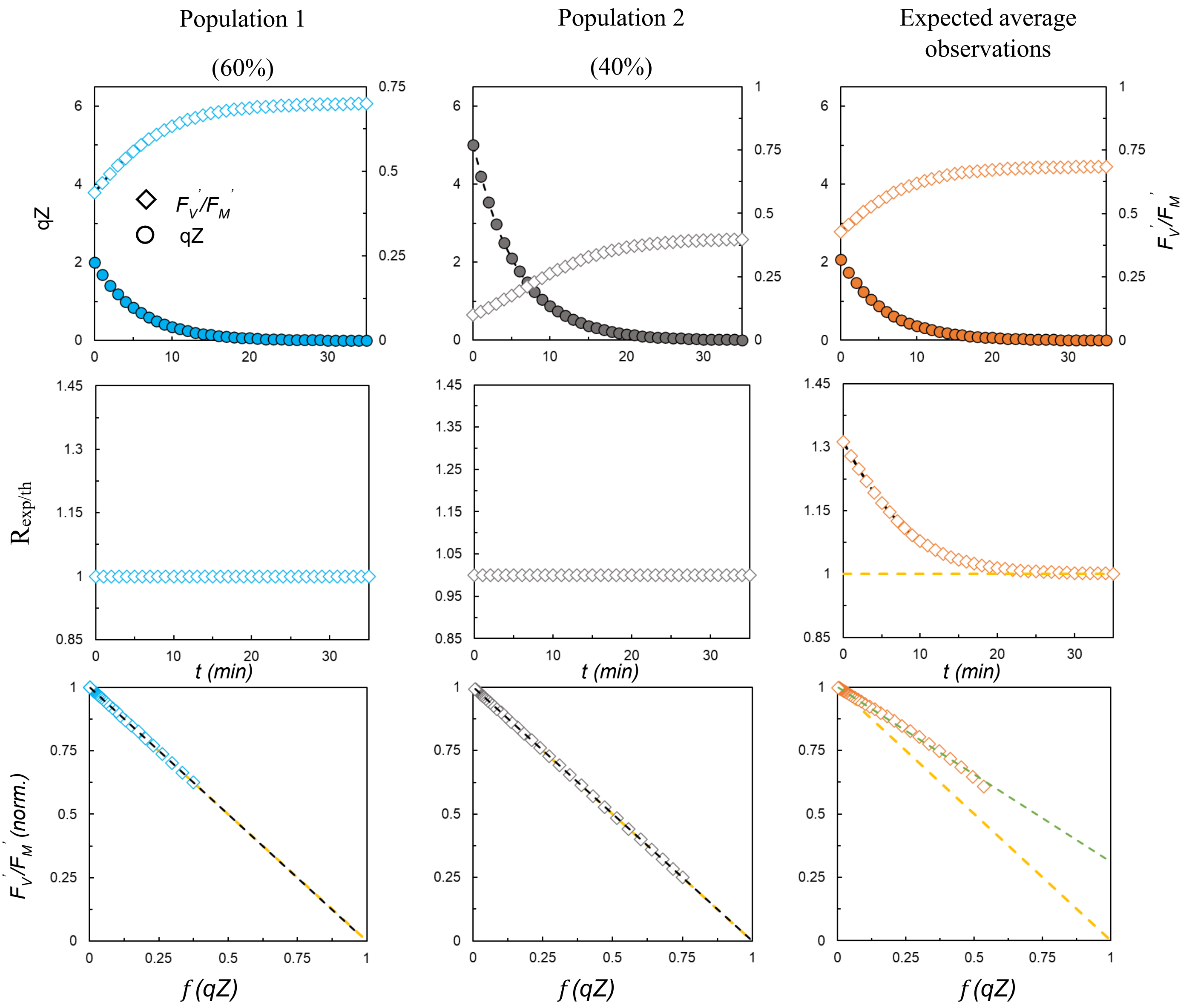

t	$F_0 1$	$F_M'1$	NPQ1	$F_V'/F_M'1$	$R_{exp/th}$ 1	$F_0 2$	$F_M'2$	NPQ2	$F_V'/F_M'2$	$R_{exp/th}$ 2	F_0Av.	F_M'Av.	NPQobs.	F_V'/F_M'(SV)	F_V'/F_M'(obs.)	$R_{exp/th}$ obs.
**Initial**	**0.30**	**1.00**		**0.70**		**0.6**	**1.00**		**0.40**		**0.42**	**1.00**		**0.58**		
0	0.19	0.33	2.00	0.44	1.00	0.15	0.17	5.00	0.10	1.00	0.17	0.27	2.75	0.27	0.35	1.31
1	0.20	0.37	1.68	0.47	1.00	0.17	0.19	4.20	0.11	1.00	0.19	0.30	2.33	0.29	0.38	1.28
2	0.21	0.41	1.41	0.49	1.00	0.19	0.22	3.54	0.13	1.00	0.20	0.34	1.97	0.32	0.40	1.25
3	0.22	0.46	1.19	0.52	1.00	0.22	0.25	2.97	0.14	1.00	0.22	0.37	1.67	0.34	0.42	1.22
4	0.23	0.50	1.00	0.54	1.00	0.24	0.29	2.50	0.16	1.00	0.23	0.41	1.41	0.36	0.43	1.19
...																

Text S2: Scenario of 95/5% populations, typical of 40 h dark acclimation (Fig. 6)

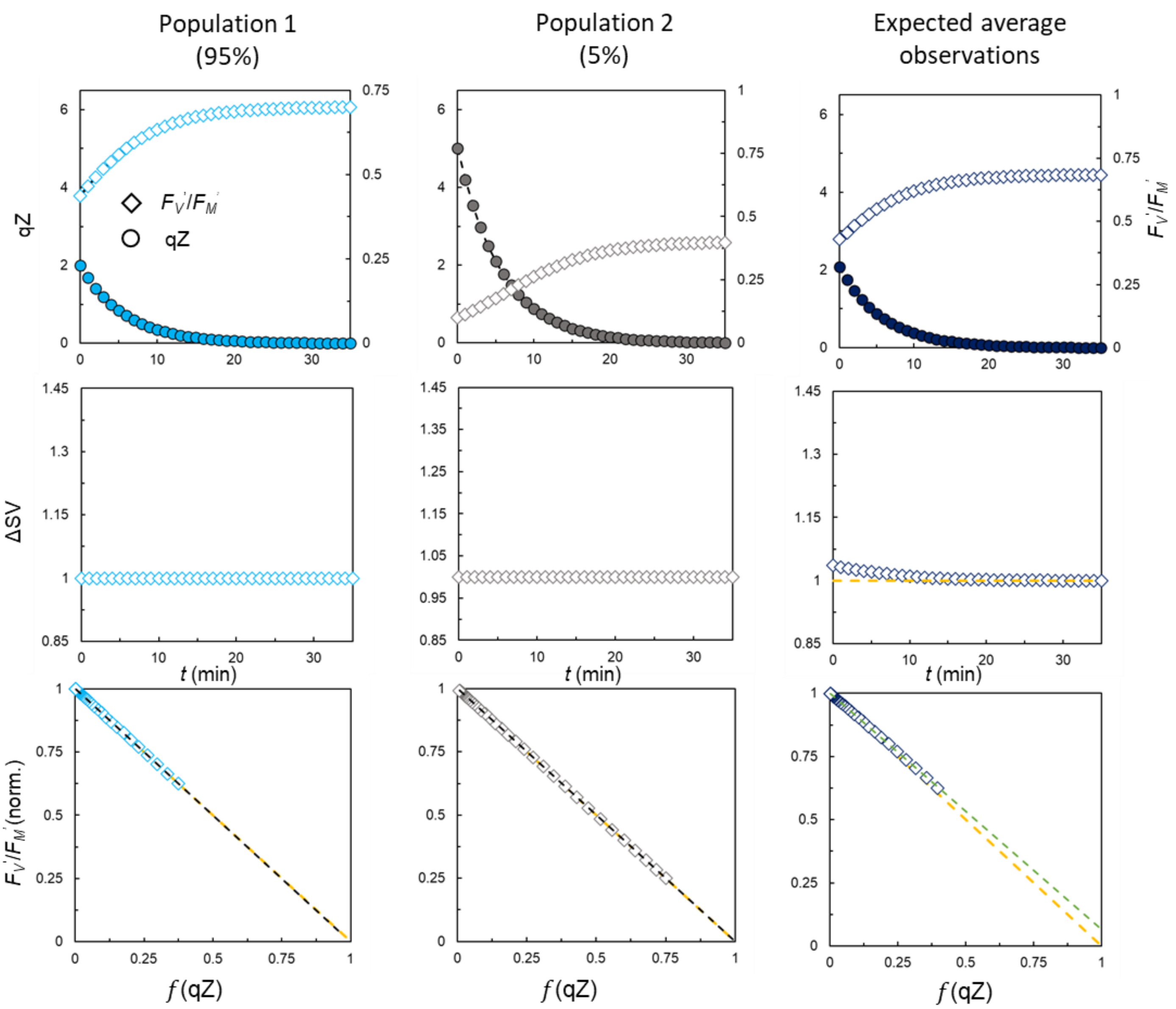

t	$F_0$1	F_M'1	NPQ1	F_V'/F_M'1	$R_{exp/th}$ 1	$F_0$2	F_M'2	NPQ2	F_V'/F_M'2	$R_{exp/th}$ 2	F_0Av.	F_M'Av.	NPQobs.	F_V'/F_M'(SV)	F_V'/F_M'(obs.)	$R_{exp/th}$ obs.
**Initial**	**0.30**	**1.00**		**0.70**		**0.6**	**1.00**		**0.40**		**0.32**	**1.00**		**0.69**		
0	0.19	0.33	2.00	0.44	1.00	0.15	0.17	5.00	0.10	1.00	0.19	0.33	2.08	0.27	0.35	1.04
1	0.20	0.37	1.68	0.47	1.00	0.17	0.19	4.20	0.11	1.00	0.20	0.36	1.75	0.29	0.38	1.03
2	0.21	0.41	1.41	0.49	1.00	0.19	0.22	3.54	0.13	1.00	0.21	0.40	1.47	0.32	0.40	1.03
3	0.22	0.46	1.19	0.52	1.00	0.22	0.25	2.97	0.14	1.00	0.22	0.45	1.24	0.34	0.42	1.03
4	0.23	0.50	1.00	0.54	1.00	0.24	0.29	2.50	0.16	1.00	0.23	0.49	1.04	0.36	0.43	1.02
...																

References

1. Lavergne, J. & Trissl, H. W. Theory of fluorescence induction in photosystem II: derivation of analytical expressions in a model including exciton-radical-pair equilibrium and restricted energy transfer between photosynthetic units. *Biophys. J.* **68**, 2474–2492 (1995).
2. Kramer, D. M., Johnson, G., Kiirats, O. & Edwards, G. E. New fluorescence parameters for the determination of QA redox state and excitation energy fluxes. *Photosynth. Res.* **79**, 209–218 (2004).
3. Stirbet, A. & Govindjee. On the relation between the Kautsky effect (chlorophyll a fluorescence induction) and Photosystem II: Basics and applications of the OJIP fluorescence transient. *Journal of Photochemistry and Photobiology B: Biology* **104**, 236–257 (2011).
4. Giovagnetti, V. & Ruban, A. V. Detachment of the fucoxanthin chlorophyll a/c binding protein (FCP) antenna is not involved in the acclimative regulation of photoprotection in the pennate diatom *Phaeodactylum tricornutum*. *Biochim. Biophys. Acta - Bioenerg.* **1858**, 218–230 (2017).
5. Lavaud, J. *et al.* Influence of the Diadinoxanthin Pool Size on Photoprotection in the Marine Planktonic Diatom *Phaeodactylum tricornutum* 1. *Plant Physiol.* **129**, 1398–1406 (2002).
6. Buck, J. M. *et al.* Lhcx proteins provide photoprotection via thermal dissipation of absorbed light in the diatom *Phaeodactylum tricornutum*. *Nat. Commun.* **10**, 4167 (2019).
7. Gorbunov, M. Y., Kolber, Z. S., Lesser, M. P. & Falkowski, P. G. Photosynthesis and photoprotection in symbiotic corals. *Limnol. Oceanogr.* **46**, 75–85 (2001).
8. Mauzerall, D. & Greenbaum, N. L. The absolute size of a photosynthetic unit. *Biochim. Biophys. Acta (BBA)-Bioenergetics* **974**, 119–140 (1989).
9. Huysman, M. J. *et al.* Genome-wide analysis of the diatom cell cycle unveils a novel type of cyclins involved in environmental signaling. *Genome Biol.* **11**, R17 (2010).
10. Annunziata, R. *et al.* Trade-off between sex and growth in diatoms: Molecular mechanisms and demographic implications. *Sci. Adv.* **8**, 1–17 (2022).
