# Supplementary material for: Pennate diatoms make non-photochemical quenching as simple as possible but not simpler
Source: Nat Commun. 2025 Mar 10;16:2385. doi: 10.1038/s41467-025-57298-4 (PMC11894083; doi:10.1038/s41467-025-57298-4)
Supplement: Supplementary file 3 — Description of Additional Supplementary Files [file 41467_2025_57298_MOESM3_ESM.pdf]

Description of Additional Supplementary Files

Supplementary Data 1: Mean ± SD and the number of replicates measured across all strains and growth conditions used to run linear regressions on baseline physiology.
