# Supplementary material for: Pennate diatoms make non-photochemical quenching as simple as possible but not simpler
Source: Nat Commun. 2025 Mar 10;16:2385. doi: 10.1038/s41467-025-57298-4 (PMC11894083; doi:10.1038/s41467-025-57298-4)
Supplement: Supplementary file 5 — Reporting Summary [file 41467_2025_57298_MOESM5_ESM.pdf]

nature portfolio

Corresponding author(s): Benjamin Bailleul

Last updated by author(s): Jul 26, 2024

Reporting Summary

Nature Portfolio wishes to improve the reproducibility of the work that we publish. This form provides structure for consistency and transparency in reporting. For further information on Nature Portfolio policies, see our Editorial Policies and the Editorial Policy Checklist.

Statistics

For all statistical analyses, confirm that the following items are present in the figure legend, table legend, main text, or Methods section.

n/a	Confirmed	
☐	☒	The exact sample size (*n*) for each experimental group/condition, given as a discrete number and unit of measurement
☐	☒	A statement on whether measurements were taken from distinct samples or whether the same sample was measured repeatedly
☐	☒	The statistical test(s) used AND whether they are one- or two-sided<br>*Only common tests should be described solely by name; describe more complex techniques in the Methods section.*
☒	☐	A description of all covariates tested
☒	☐	A description of any assumptions or corrections, such as tests of normality and adjustment for multiple comparisons
☐	☒	A full description of the statistical parameters including central tendency (e.g. means) or other basic estimates (e.g. regression coefficient) AND variation (e.g. standard deviation) or associated estimates of uncertainty (e.g. confidence intervals)
☐	☒	For null hypothesis testing, the test statistic (e.g. *F*, *t*, *r*) with confidence intervals, effect sizes, degrees of freedom and *P* value noted<br>*Give P values as exact values whenever suitable.*
☒	☐	For Bayesian analysis, information on the choice of priors and Markov chain Monte Carlo settings
☒	☐	For hierarchical and complex designs, identification of the appropriate level for tests and full reporting of outcomes
☒	☐	Estimates of effect sizes (e.g. Cohen's *d*, Pearson's *r*), indicating how they were calculated

Our web collection on statistics for biologists contains articles on many of the points above.

Software and code

Policy information about availability of computer code

Data collection	No software was used to collect data.
Data analysis	Most data was analyzed using R-4.4.0. MACSQantify 3.0.1 was used for cytometer data and Origin 9.1 was used for specific fitting purpose when mentioned in the manuscript. Image lab v5.0 was also used.

For manuscripts utilizing custom algorithms or software that are central to the research but not yet described in published literature, software must be made available to editors and reviewers. We strongly encourage code deposition in a community repository (e.g. GitHub). See the Nature Portfolio guidelines for submitting code & software for further information.

Data

Policy information about availability of data

All manuscripts must include a data availability statement. This statement should provide the following information, where applicable:

- Accession codes, unique identifiers, or web links for publicly available datasets
- A description of any restrictions on data availability
- For clinical datasets or third party data, please ensure that the statement adheres to our policy

Data will be deposited on Dryad before publication of this manuscript.

Research involving human participants, their data, or biological material

Policy information about studies with human participants or human data. See also policy information about sex, gender (identity/presentation), and sexual orientation and race, ethnicity and racism.

Reporting on sex and gender	NA
Reporting on race, ethnicity, or other socially relevant groupings	NA
Population characteristics	NA
Recruitment	NA
Ethics oversight	NA

Note that full information on the approval of the study protocol must also be provided in the manuscript.

Field-specific reporting

Please select the one below that is the best fit for your research. If you are not sure, read the appropriate sections before making your selection.

☒ Life sciences ☐ Behavioural & social sciences ☐ Ecological, evolutionary & environmental sciences

For a reference copy of the document with all sections, see nature.com/documents/nr-reporting-summary-flat.pdf

Life sciences study design

All studies must disclose on these points even when the disclosure is negative.

Sample size	NA
Data exclusions	No data was exluded.
Replication	All experiments have been replicated 3 to 14 times (see Table 1 and S1 for details about the number of replicates). The experiments showing the highest variability (as discussed in the manuscript) were that following the 40 hours dark incubation, which were repeated 8 times with 5 different strains.
Randomization	NA
Blinding	NA

Reporting for specific materials, systems and methods

We require information from authors about some types of materials, experimental systems and methods used in many studies. Here, indicate whether each material, system or method listed is relevant to your study. If you are not sure if a list item applies to your research, read the appropriate section before selecting a response.

Materials & experimental systems

n/a	Involved in the study	
☐	☒	Antibodies
☒	☐	Eukaryotic cell lines
☒	☐	Palaeontology and archaeology
☒	☐	Animals and other organisms
☒	☐	Clinical data
☒	☐	Dual use research of concern
☒	☐	Plants

Methods

n/a	Involved in the study	
☒	☐	ChIP-seq
☐	☒	Flow cytometry
☒	☐	MRI-based neuroimaging

Antibodies

Antibodies used	Rabbit polyclonal anti-LHCSR antibody (K. K. Niyogi, University of California), ATP-B; AS05085, Agrisera, Sweden, anti-D2 (J-D Rochaix, University of Geneva)

Validation	- Rabbit polyclonal anti-LHCSR antibody (K. K. Niyogi, University of California): Bailleul et al, PNAS 2010; Taddei et al, Plant Phys 2018, Lepetit et al, Plant Phys 2013.<br><br>- ATP-B; AS05085, Agrisera, Sweden: https://www.agrisera.com/en/artiklar/atpb-beta-subunits-of-atp-synthase-global-antibody.html<br><br>- D2 (J-D Rochaix, University of Geneva): Bailleul et al, PNAS 2010; Taddei et al, JXB 2016; De Riso et al, NAR 2009.

Plants

Seed stocks	NA
Novel plant genotypes	NA
Authentication	NA

Flow Cytometry

Plots

Confirm that:

- [x] The axis labels state the marker and fluorochrome used (e.g. CD4-FITC).
- [x] The axis scales are clearly visible. Include numbers along axes only for bottom left plot of group (a 'group' is an analysis of identical markers).
- [ ] All plots are contour plots with outliers or pseudocolor plots.
- [x] A numerical value for number of cells or percentage (with statistics) is provided.

Methodology

Sample preparation	Sample were prepared as described in material and methods
Instrument	MACSQuant Analyser flow cytometer (Miltenyi Biotec, Germany)
Software	MACSQantify 3.0.1 and R-4.4.0
Cell population abundance	Pior to fixing cell in EtOH, cell population were confirmed to be between 5*10^5 and 1*10^6 with a particle counter.
Gating strategy	We use one-dimension histogram to access the distribution of DAPI-fluorescence per cell (therefore not all cytometer plot are "contour plots", but contour plots will be provided in supplemental if requested by the editor. We manually set the gate at the intersection between the main and secondary cell population(s) to estimate the proportion of cells undergoing division in the culture.

- [x] Tick this box to confirm that a figure exemplifying the gating strategy is provided in the Supplementary Information.
